# Epidemiological trends of mechanically ventilated acute respiratory distress syndrome in the twenty-first century: a nationwide, population-based retrospective study

**DOI:** 10.1186/s40560-025-00781-3

**Published:** 2025-02-17

**Authors:** Miguel Bardají-Carrillo, Rocío López-Herrero, Gerardo Aguilar, Irene Arroyo-Hernantes, Esther Gómez-Sánchez, Luigi Camporota, Jesús Villar, Eduardo Tamayo

**Affiliations:** 1 BioCritic, Group for Biomedical Research in Critical Care Medicine, 47003 Valladolid, Spain; 2 Anesthesiology and Critical Care, Clinical University Hospital of Valladolid, Av. Ramón y Cajal, 3, 47003 Valladolid, Spain; 3 CIBER de Enfermedades Infecciosas (CIBERINFEC), Instituto de Salud Carlos III, Madrid, Spain; 4 Department of Surgery, University of Valladolid, 47003 Valladolid, Spain; 5 Department of Anesthesiology and Intensive Care, Surgical Intensive Care Unit, Hospital Clínico Universitario de Valencia, Avenida Blasco Ibáñez, 17, 46010 Valencia, Spain; 6 INCLIVA, Institute of Research, Avenida Blasco Ibáñez, 17, 46010 Valencia, Spain; 7 School of Medicine, University of Valencia, Avenida Blasco Ibáñez, 15, 46010 Valencia, Spain; 8 Department of Research and Innovation, Clinical University Hospital of Valladolid (HCUV), SACYL/IECSCYL, 47003 Valladolid, Spain; 9 Department of Adult Critical Care, Guy’s and St Thomas’ NHS Foundation Trust, London, UK; 10 Centre for Human and Applied Physiological Sciences, King’s College London, London, UK; 11 CIBER de Enfermedades Respiratorias, Instituto de Salud Carlos III, Madrid, Spain; 12 Research Unit at Hospital Universitario Dr. Negrín, Fundación Canaria Instituto de Investigación Sanitaria de Canarias, Barranco de la Ballena s/n, 35019 Las Palmas de Gran Canaria, Spain; 13 Li Ka Shing Knowledge Institute at St. Michael’s Hospital, Toronto, ON Canada; 14 Faculty of Health Sciences, Universidad del Atlántico Medio, Tafira Baja, Las Palmas, Spain

**Keywords:** Acute respiratory distress syndrome, Epidemiology, Minimum Basic Data Set, Incidence, Mechanical ventilation, Mortality, Health care cost

## Abstract

**Purpose:**

Acute respiratory distress syndrome (ARDS) is a prevalent respiratory condition associated with significant mortality. Current literature on ARDS epidemiology reports a wide range of incidence (7.2–78.9/100,000 population/year), hospital mortality (32–51%), and associated costs ($8476–$547,974). We have analyzed epidemiological trends of mechanically ventilated ARDS (MV-ARDS) in Spain from 2000 to 2022 using the Minimum Basic Data Set (MBDS), focusing on MV-ARDS incidence, associated mortality, and economic impact.

**Methods:**

We conducted a nationwide, population-based retrospective study of all hospitalizations for MV-ARDS in Spanish hospitals—from January 1, 2000 to December 31, 2022—using MBDS records, with an estimated coverage of 99.5%. The study reports MV-ARDS incidence per 100,000 population/year, hospital mortality rate, and mean cost per patient. We also considered the effect of COVID-19 on MV-ARDS epidemiology.

**Results:**

We analyzed 93,192 records of patients with a new diagnosis of MV-ARDS during the study period. MV-ARDS incidence ranged from 2.96 to 20.14/100,000 population-years, peaking in 2021. Mortality ranged between 38.0 and 55.0%, showing a declining trend, while the cost per patient increased, stabilizing ~€30,000–€40,000 after reaching a peak of €42,812 in 2011. During the COVID-19 pandemic, hospital stay lengthened (*p* < 0.001), while hospital mortality decreased (*p* < 0.001). There was an increased proportion of patients with obesity and diabetes mellitus, with fungal or viral etiologies.

**Conclusion:**

This is the largest epidemiological study on ARDS in Europe. MV-ARDS incidence has stabilized in recent years, with mortality showing a declining trend. ARDS-related costs have increased nearly fourfold. MBDS data could enhance ARDS understanding and guide future studies.

**Supplementary Information:**

The online version contains supplementary material available at 10.1186/s40560-025-00781-3.

## Background

Acute respiratory distress syndrome (ARDS) is a heterogeneous clinical condition characterized by an acute onset of respiratory failure [1]. It poses a significant global health challenge due to its high clinical prevalence and associated mortality rates [2].

Epidemiological data are fundamental in clinical research, offering valuable insights into disease pathogenesis, improving diagnostic accuracy, and in identifying, mitigating, or reverting treatable risk factors [3]. However, obtaining accurate epidemiological data for ARDS has been challenging [4] due to its complex and changing clinical definition [1, 5, 6], and the geographic variations in recognition and reporting [6].

The reported incidence of ARDS varies widely in the literature, ranging between 7.2 and 78.9 cases per 100,000 population/year, with a prevalence between 7.1 and 19.0% among Intensive Care Units (ICU) admissions. [7], while the PANDORA study revealed that ARDS represented 3.4% of ICU admissions, and 7.5% of patients who received mechanical ventilation (MV) [8]. Similarly, ARDS mortality rates are highly variable, with hospital mortality ranging from 32 to 51%. Since 2010, the reported mortality rates have been 45% for hospital mortality, 38% for ICU mortality, 30% for 28- or 30-day mortality, and 32% for 60-day mortality [9]. The associated costs of ARDS also varied, ranging between $8476 and $547,974 [10], with no studies assessing current ARDS costs in Europe. Furthermore, the impact of COVID-19 on ARDS epidemiology remains unreported [11].

Long-term clinical studies on ARDS are limited, often reflecting practices in selected centers that are not representative of the wider range of ICUs worldwide, and they fail to provide a reliable epidemiological profile of ARDS based on robust clinical outcomes [12]. Although administrative databases like the Minimum Basic Data Set (MBDS) have been underutilized in ARDS research, they offer a wealth of useful data [12]. Given the scarcity of MBDS-based epidemiological studies on ARDS, the variability in ARDS epidemiology, and the lack of economical assessment in Europe, this study aims to perform a nationwide analysis of the epidemiological trends of incidence, hospital mortality, and associated costs in Spain from 2000 to 2022. We also assessed the effects of COVID-19 on ARDS epidemiology.

## Methods

### Study design and data source

We conducted a nationwide, population-based retrospective study of all hospitalizations with a diagnosis of ARDS in any hospital in Spain between January 1, 2000, and December 31, 2022. It should be noted that before 2016, the MBDS included only public hospitals, whereas from 2016 onwards, it covered both public and private hospitals.

The year 2016 was excluded from the analysis because the Spanish Ministry of Health was unable to provide data for that year due to the implementation of a new data model for the MBDS [13]. We have analyzed only ARDS patients who received invasive MV.

Clinical and administrative data were sourced from hospital records in the MBDS of the National Surveillance System for Hospital Data in Spain. MBDS data, provided by the Ministry of Health, are published annually with a 2-year delay. The MBDS is a comprehensive clinical and administrative database that includes clinical information recorded at the time of hospital discharge, with an estimated coverage of 99.5% [13, 14], so the vast majority of hospitals and patients are recorded in this database, giving consistency to the data. The MBDS includes up to 20 diagnoses, each indicating whether the diagnosis was present on admission, and 20 therapeutic procedures performed during the hospital stay. According to the Spanish legislation, the MBDS provides de-identified, encrypted patient identification numbers, ensuring that the identification of individual patients is not possible, gender, date of birth, dates of hospital admission and discharge, medical institutions providing the services, the diagnosis and procedure codes, or the outcome at discharge, according to the *International Classification of Diseases 9th Revision, Clinical Modification* (ICD-9-CM) [15] or to the *International Classification of Diseases 10th Revision, Clinical Modification* (ICM-10-CM) [16] depending on the period of the study.

The MBDS is governed by legislation that outlines how institutions must handle health-related personal data. It is important to note that when working with MBDS data, the dataset may not always contain all the clinical details that could be needed, such as chest radiographs, arterial oxygen partial pressure to fractional inspired oxygen ratio (for grading ARDS severity). This study was approved by the Ethics Committee of Valladolid East Health Area under the code PI-24–399-C. Given the anonymous and mandatory nature of data, informed consent was waived.

### Study variables

We selected all patients hospitalized in all hospitals in Spain with a diagnosis of respiratory distress syndrome (ICD-9-CM codes 518.82 and 518.5, and ICD-10-CM codes J80.*) and the need for invasive MV (ICD-9 codes 96.70, 96.71, 96.72, 96.04, and ICD-10 codes 5A1935Z, 5A1945Z, 5A1955Z). Using these invasive MV codes, we also evaluated MV duration, with no exclusion criteria applied.

Sepsis was defined using the codes adapted from MacLaren et al. [17], Esper et al. [18], Dombrovskiy et al. [19], and Bateman et al. [20] (Supplementary Tables S1, S2). The presence of an infection source (Supplementary Tables S5, S6) was defined using the codes adapted from Esper et al. [18] and Wang et al. [21], and organ dysfunction (Supplementary Tables S3, S4) according to sepsis criteria by Angus et al. [22] adapted by Shen et al. [23] and Bateman et al. [20]. All codes were updated to ICD-10-CM by our group [24].

Demographic data included age, sex, comorbidities (diabetes, hypertension, heart disease, chronic renal disease, respiratory disease, neurological disease), infection and site of infection, length of hospital stay, and mortality. The study was divided into two periods, due to the change in ARDS codification from ICD-9-CM to ICD-10-CM between 2015 and 2016. The primary outcomes were mechanically ventilated ARDS (MV-ARDS) incidence per 100.000 population/year, MV-ARDS hospital mortality rate, and mean cost per MV-ARDS patient. The secondary outcomes included length of hospital stay (LOHS), site of infection, comorbidities assessment, and the effects of COVID-19 on the epidemiology of MV-ARDS.

### Statistical analysis

The incidence of MV-ARDS was defined as the number of cases per 100.000 inhabitants in the population. The percentage of MV-ARDS hospital mortality was defined as the proportion of overall in-hospital deaths in MV-ARDS patients. To further assess the effect of COVID-19 on MV-ARDS epidemiology, pre-pandemic period included patients diagnosed from 01/01/2017 to 29/02/2020, intra-pandemic period included patients from 01/03/2020 to 28/02/2022, and post-pandemic period included patients from 01/03/2022 to 31/12/2022. Differences between groups were assessed using the Chi-square test for categorical variables and the ANOVA test for continuous variables normally distributed and Kruskal–Wallis test for continuous variables not normally distributed. Kolmogorov–Smirnov test was used to assess the normality of the variables. Categorical variables were expressed in percentages, while continuous variables were expressed as mean ± standard deviation (SD). Multivariable Poisson regression analysis was performed to evaluate temporal trends in the incidence, hospital mortality, and associated costs of MV-ARDS, adjusting for age and sex. The LOHS was obtained as the difference in days between date of hospital admission and date of discharge or death. Hospital admission day was considered day 0. Discharge on the same day was considered 1-day stay. Costs were calculated using diagnosis-related groups (DRG), which represents a medical-economic entity concerning a set of diseases requiring analogous management resources. DRG data were extracted from the MBDS. All analyses were performed using the R statistical package, version 4.3.2. All tests conducted were two-tailed, and *p* values < 0.05 were considered statistically significant.

## Results

### Patients’ characteristics

Table 1 shows the characteristics of patients. A total of 93,192 of patient records were diagnosed with MV-ARDS between 2000 and 2022: 68,213 patients between 2000 and 2015, and 24,979 patients between 2017 and 2022. MV-ARDS was more frequent in men (65.1% in 2000–2015 and 68.3% in 2017–2022), and the mean age was 58 years (2000–2015) and 60 years (2017–2022). Mean length of hospital stay was 35.7 (41.4) days in 2000–2015 and 37.6 (33.7) days in 2017–2022. The most frequent etiology was respiratory tract infection, appearing in 42.0% of patients in the first period and in 78.5% during the second period. Sepsis was the most common comorbidity between 2000 and 2015 (66.1%), whereas in the second period, COVID-19 (65.8%) was the most prevalent, followed by sepsis (43.7%). ECMO use increased during the second period of the study compared to the first. When evaluating MV duration across the two periods, patients in both groups were more likely to require MV for over 96 h (54.4% in the CIE-9 period and 79.9% in the CIE-10 period). In addition, during the ICD-9 period, 20% of patients were assigned the 96.70 code, indicating an unspecified duration of MV.

**Table 1:** Patient’s characteristics

	2000–2015 (*n* = 68,213)	2017–2022 (*n* = 24,979)	*p* value
*Characteristics*			
Sex (male) [% (*n*)]	65.1% (44,392)	68.3% (17,064)	**<0.001**
Age (years) [mean (SD)]	58.5 (20.46)	60.3 (14.9)	**<0.001**
Medical condition vs. surgical condition [% (*n*)]	53.4% (36,393)	64.9% (16,198)	**<0.001**
Charlson index [mean (SD)]	0.8 (0.92)	0.5 (0.90)	**<0.001**
*Comorbidities [% (n)]*			
Diabetes mellitus	10.1% (6913)	21.0% (5254)	**<0.001**
Obesity	4.9% (3313)	18.7% (4668)	**<0.001**
Chronic respiratory diseases	12.3% (8375)	13.3% (3323)	**<0.001**
Arterial hypertension	20.4% (13,880)	33.0% (8239)	**<0.001**
Ischemic heart diseases	5.7% (3871)	1.9% (474)	**<0.001**
Cancer	19.1% (13,042)	8.7% (2178)	**<0.001**
HIV	1.2% (849)	0.8% (188)	**<0.001**
Hepatic diseases	4.1% (2777)	6.5% (1619)	**<0.001**
Renal diseases	14.6% (9972)	6.7% (1672)	**<0.001**
Aspergillosis	0.9% (603)	4.1% (1022)	**<0.001**
Influenza	1.5% (998)	1.7% (427)	**0.007**
COVID-19	0.00% (0)	65.8% (16,442)	
*Sites of infection [% (n)]*			
Central nervous system	1.1% (753)	0.3% (66)	**<0.001**
Circulatory	0.7% (504)	0.8% (190)	0.786
Digestive	13.7% (9371)	5.3% (1333)	**<0.001**
Genitourinary	8.8% (6016)	20.2% (5048)	**<0.001**
Respiratory	42.0% (28,634)	78.5% (19,597)	**<0.001**
Skin	2.8% (1874)	1.7% (435)	**<0.001**
Others	19.5% (13,322)	17.7% (4419)	**<0.001**
*Outcomes*			
Sepsis [% (*n*)]	66.1% (45,097)	43.7% (10,915)	**<0.001**
MV < 96 h [% (*n*)]	25.5% (17,403)	21.1% (5055)	**<0.001**
MV > 96 h [% (*n*)]	54.5% (37,198)	79.9% (19,924)	**<0.001**
ECMO [% (*n*)]	0.26% (179)	0.4% (148)	**<0.001**
LOHS (days) [mean (SD)]	35.7 (41.4)	37.6 (33.67)	**<0.001**
In-hospital mortality [% (*n*)]	48.5% (33,111)	43.1% (10,753)	**<0.001**

Continuous variables are represented as mean and standard deviation (SD); categorical variables are represented as percentages (%) and number (*n*).* p* values in bold indicate statistical significance

*MV* mechanical ventilation, *ECMO* extracorporeal membrane oxygenation, *LOHS* length of hospital stay

### MV-ARDS incidence

**Fig. 1:**
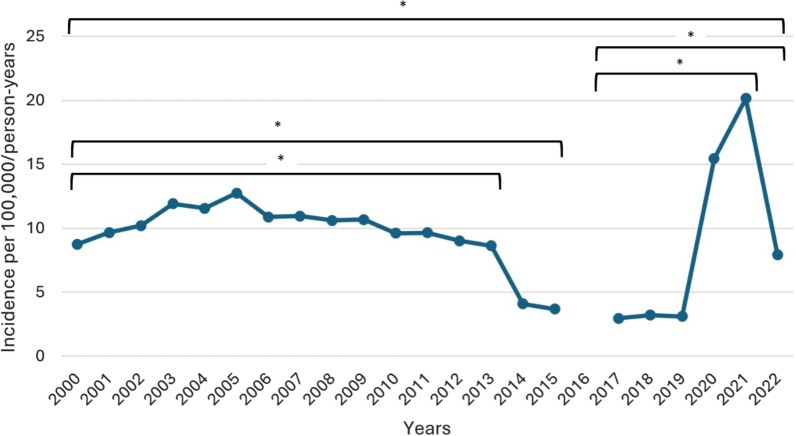
Evolution of MV-ARDS incidence per 100,000 person-years. * indicates that the difference in incidence between the beginning and end of the period is statistically significant (*p* < 0.05). *p* values were two-tailed and calculated using multivariable Poisson regression

**Fig. 2:**
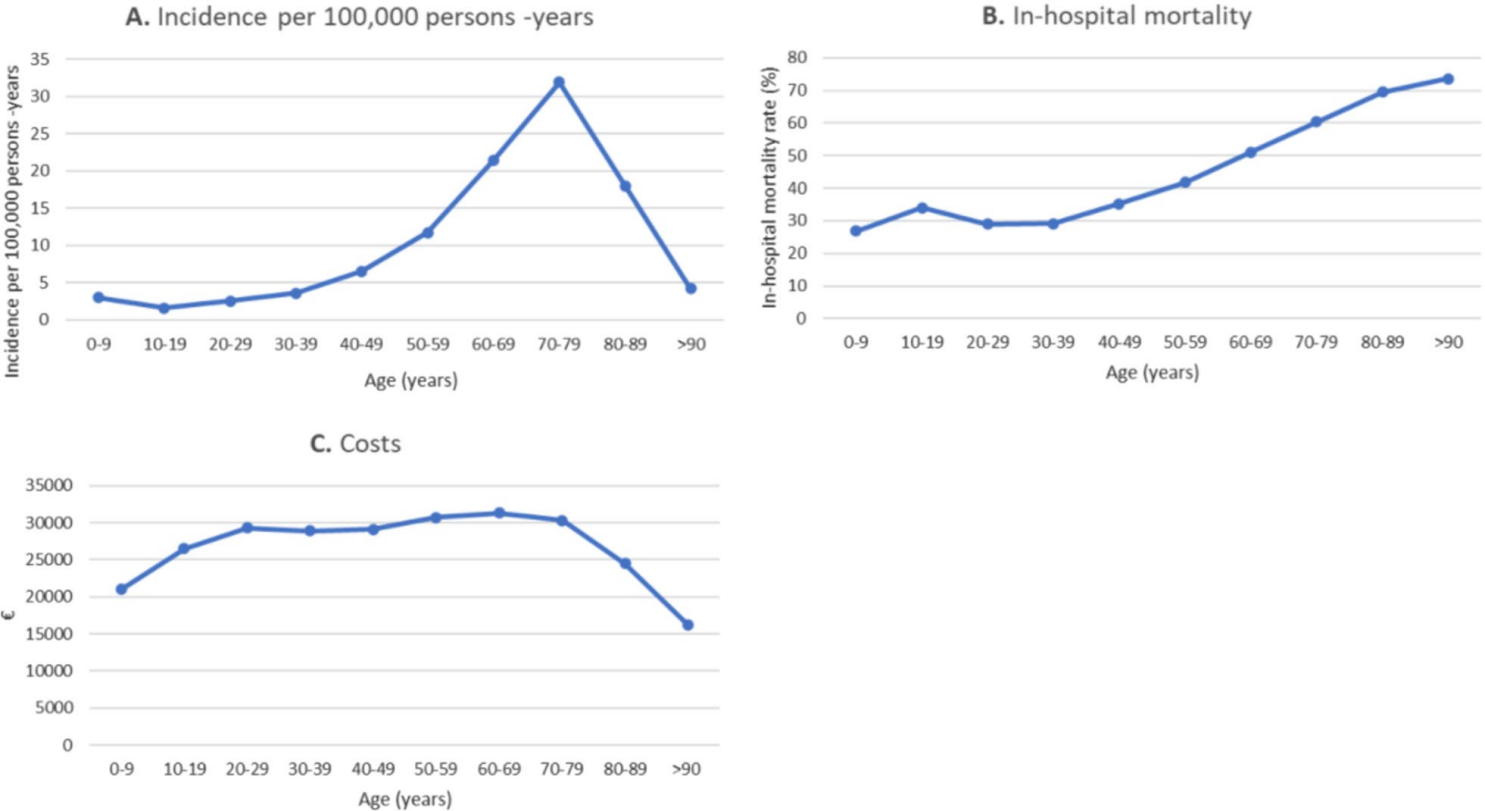
**A** Incidence per 100,000 persons-years by age groups; **B** in-hospital mortality by age groups; **C** costs by age groups

The variation in MV-ARDS incidence per 100,000 population per year is shown in Fig. 1. The incidence varied between 2.96/100,000 cases and 20.14/100,000 cases, reaching the peak in 2021 (20.14/100,000). Between 2000 and 2013 ARDS, incidence showed lower variability. However, from 2014 until 2019, there was a significant decrease, followed by a sharp increase in 2020–2022 that surpassed the previous incidence rates, very likely related to the COVID-19 pandemics. The incidence differed significantly between 2000–2013, 2000–2015, 2000–2022, 2017–2021, and 2017–2022, according to the Poisson regression analysis. MV-ARDS incidence by age group is depicted in Fig. 2A.

### MV-ARDS in-hospital mortality

**Fig. 3:**
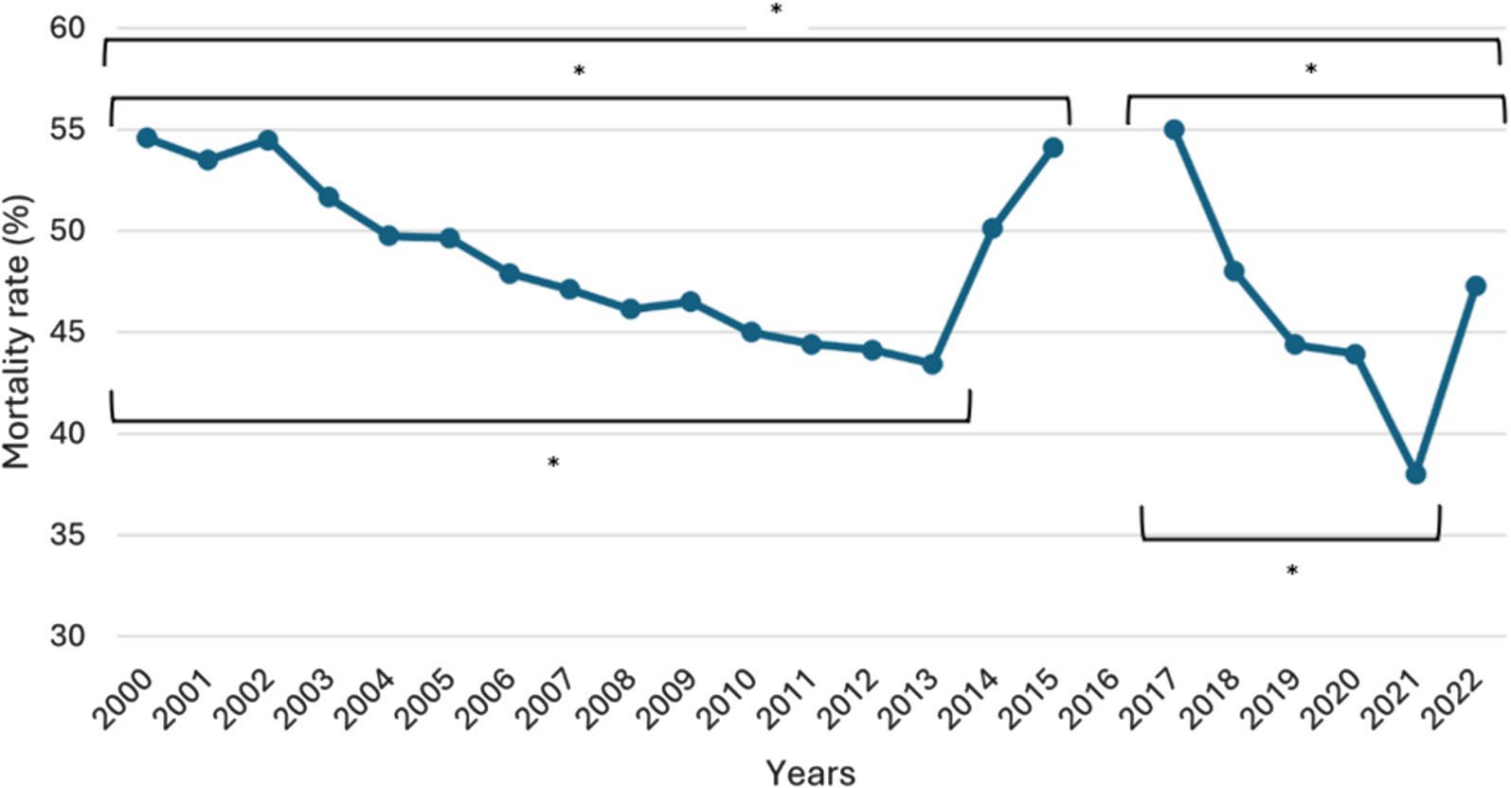
Evolution of in-hospital mortality rates for MV-ARDS patients. * indicates that the difference in incidence between the beginning and end of the period is statistically significant (*p* < 0.05). *p* values were two-tailed and calculated using multivariable Poisson regression

The trend in hospital mortality for MV-ARDS patients, ranging between 38.0 and 55.0%, is illustrated in Fig. 3, with an average mortality of 48.5% during the first period and 43.1% in the second period (Table 1). The trend shows a decrease in MV-ARDS mortality. Hospital mortality differed significantly between the periods 2000–2013, 2000–2015, 2000–2022, 2017–2021, and 2017–2022, according to the Poisson regression analysis. MV-ARDS hospital mortality by age group is depicted in Fig. 2B.

### Mean cost per MV-ARDS patient

**Fig. 4:**
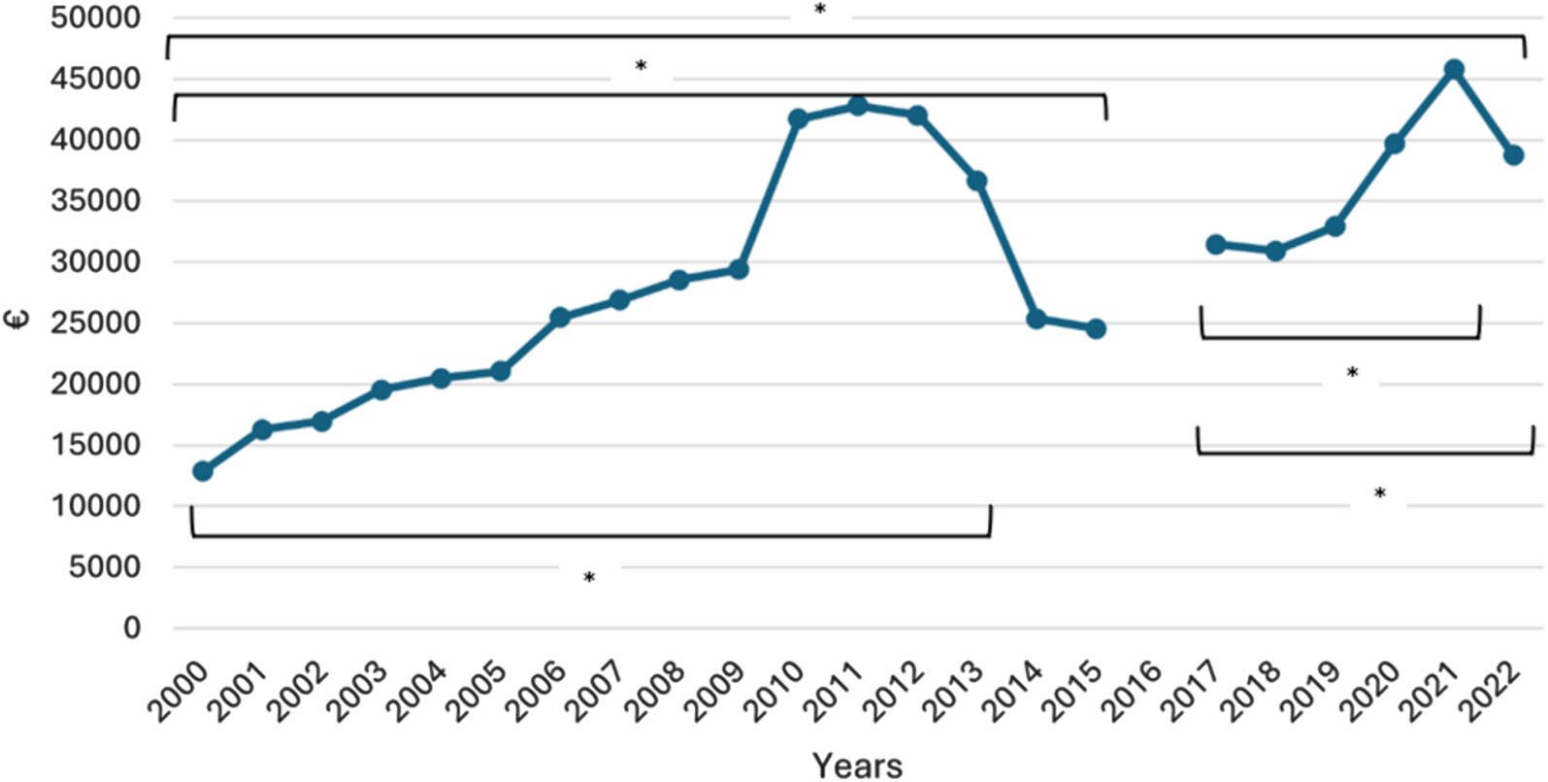
Evolution of mean cost per MV-ARDS patient, expressed in €. * indicates that the difference in incidence between the beginning and end of the period is statistically significant (*p* < 0.05). *p* values were two-tailed and calculated using multivariable Poisson regression

Mean health care cost per MV-ARDS patient between 2000 and 2022 is depicted in Fig. 4. The trend revealed a steady increment. In 2000, the mean cost per MV-ARDS patient was €12,854.91, which rose to €42,811.59 by 2011, before subsequently stabilizing between €30,000 and €40,000 per patient. The costs differed significantly between 2000–2013, 2000–2015, 2000–2022, 2017–2021, and 2017–2022, according to the Poisson regression analysis. MV-ARDS costs by age group are depicted in Fig. 2C.

### Effect of COVID-19 in MV-ARDS epidemiology

The three periods in Spain, pre-pandemic period (from 01/01/2017 to 01/03/2020), intra-pandemic period (from 01/03/2020 to 01/03/2022), and post-pandemic period (from 01/03/2022 to 31/12/2022), are compared in Table 2. During the intra-pandemic phase, length of hospital stay was longer (*p* < 0.001), but hospital mortality was lower (*p* < 0.001). Respiratory infections were present in 89% of MV-ARDS patients during the intra-pandemic period; aspergillosis increased (*p* < 0.001) whereas influenza decreased (*p* < 0.001). When evaluating the duration of MV across the three periods, more patients required MV for over 96 h. During the intra-pandemic period, a higher proportion of patients required more prolonged MV compared to the pre-pandemic or post-pandemic periods (83.3% vs. 69.3% and 70.1%, respectively).

**Table 2:** Comparison of ARDS patients between pre-pandemic period (from 01/01/2017 to 29/02/2020), intra-pandemic period (from 01/03/2020 to 28/02/2022), and post-pandemic period (from 01/03/2022 to 31/12/2022)

	Pre-pandemic (*n* = 4878)	Intra-pandemic (*n* = 18,370)	Post-pandemic (*n* = 1731)	*p* value
*Characteristics*				
Sex (male) [% (*n*)]	64.5% (3144)	69.8% (12,822)	63.4% (1098)	**<0.001**
Age (years) [mean (SD)]	57.0 (18.9)	61.2 (13.1)	59.9 (17.9)	**<0.001**
Medical condition vs. surgical condition [% (*n*)]	50.7% (2473)	69.7% (12,800)	53.4% (925)	**<0.001**
Charlson index [mean (SD)]	1.6 (1.96)	0.9 (1.34)	1.6 (1.9)	**<0.001**
*Comorbidities [% (n)]*				
Diabetes mellitus	17.0% (829)	22.4% (4117)	17.8% (308)	**<0.001**
Obesity	10.8% (528)	21.6% (3971)	9.8% (169)	**<0.001**
Chronic respiratory disease	14.2% (690)	13.0% (2394)	13.8% (239)	**<0.001**
Arterial hypertension	27.0% (1318)	35.2% (6461)	26.6% (460)	**<0.001**
Ischemic heart disease	3.3% (161)	1.4% (255)	3.4% (58)	**<0.001**
Cancer	15.5% (758)	6.1% (1112)	17.8% (308)	**<0.001**
HIV	1.8% (89)	0.5% (84)	0.9% (15)	**<0.001**
Hepatic diseases	12.9% (629)	8.1% (1491)	13.8% (238)	**<0.001**
Renal diseases	7.6% (372)	6.2% (1135)	9.5% (165)	**<0.001**
Aspergillosis	1.9% (91)	4.7% (865)	3.8% (66)	**<0.001**
Influenza	13.7% (667)	0.2% (27)	2.2% (38)	**<0.001**
COVID-19	1.5% (71)	86.1% (15,820)	27.5% (476)	**<0.001**
*Sites of infection [% (n)]*				
Central nervous system	0.6% (28)	0.2% (29)	0.5% (9)	**<0.001**
Circulatory	1.3% (63)	0.6% (109)	1.0% (18)	**<0.001**
Digestive	10.8% (529)	3.4% (627)	10.2% (177)	**<0.001**
Genitourinary	13.9% (679)	22.4% (4106)	15.2% (263)	**<0.001**
Respiratory	48.6% (2372)	89.0% (16,356)	50.2% (869)	**<0.001**
Skin	3.8% (185)	1.1% (200)	2.9% (50)	**<0.001**
Others	15.8% (769)	18.5% (3402)	14.3% (248)	**<0.001**
*Outcomes*				
Sepsis	60.1% (2929)	50.0% (9175)	57.7% (999)	**<0.001**
MV < 96 h [% (*n*)]	30.7% (1492)	16.7% (3068)	29.9% (517)	**<0.001**
MV > 96 h [% (*n*)]	69.3% (3383)	83.3% (15,302)	70.1% (1214)	**<0.001**
LOHS (days) [mean (SD)]	37.4 (37.82)	38.2 (32.83)	32.7 (29.49)	**<0.001**
In-hospital mortality [% (*n*)]	48.1% (2344)	40.7% (7472)	54.1% (937)	**<0.001**

Continuous variables are represented as mean and standard deviation (SD); categorical variables are represented as percentages (%) and number (*n*).* p* values in bold indicate statistical significance

*MV* mechanical ventilation, *LOHS* length of hospital stay

## Discussion

This study is the largest investigation into ARDS epidemiology and the first comprehensive assessment of ARDS-associated costs in a European country. In this cohort of 93,192 ARDS patients receiving MV for ARDS in Spanish hospitals between 2000 and 2022, the most relevant findings were: (1) the MV-ARDS incidence varied between 2.96/100,000 and 20.14/100,000 cases per year, peaking during COVID-19 pandemic; (2) hospital mortality rate showed a decreasing trend; (3) the average cost per patient stabilized around €30,000 and €40,000 between 2017 and 2022; (4) during the COVID-19 pandemic, comorbidities such as obesity, diabetes mellitus, and aspergillosis increased markedly.

The incidence of ARDS is variably reported in the literature, primarily as percentage of hospitalized patients or patients admitted to the ICU who develop ARDS. Most reports express the incidence as cases per 100,000 population per year. We found that the incidence of ventilated ARDS in Spain varied over the 22 years. In 2000, the incidence was 8.74/100,000; followed by a steady increase that peaked in 2005 during an influenza pandemic [25]. From 2000 to 2013, the incidence of ARDS remained relatively stable at around 10/100,000. Villar et al. [26] reported an estimated incidence of 7.2/100,000 in 2008–2009, although their study only included moderate and severe ARDS patients, while the incidence in Finland was 10.6/100,000 in 2007 [27], both studies focusing on MV-ARDS patients. After 2013, there was a subsequent decrease in ARDS diagnoses until 2019. This decline is likely attributable to the implementation of Berlin Definition criteria in 2012 [1]. Moreover, the transition from ICD-9-CM to ICD-10-CM between 2015 and 2016 probably had an impact on ARDS diagnosis, as this change has also affected the epidemiology of other clinical conditions [28, 29]. As a result, these changes could have an influence in incidence, mortality, and costs observed in our study, as fewer ARDS cases were captured by MBDS. Consequently, our data may not fully represent all ARDS patients diagnosed in Spain during the study period. Further research should evaluate the long-term impact of such transitions on epidemiological data. Lastly, during the 2020–2022 period, the COVID-19 pandemic led to a marked increase in MV-ARDS incidence in Spain, soaring to 20.14/100,000, establishing COVID-19 as a major cause of ARDS [30]. However, we could not find specific references detailing ARDS incidence per 100,000 population/year during the COVID-19 pandemic. It is important to note that MBDS data does not allow for tracking whether patients were hospitalized multiple times due to ARDS. However, since patients with an active ARDS diagnosis are unlikely to be discharged from the hospital, readmissions would likely represent new ARDS episodes. Therefore, we think that the incidence of ARDS would not be significantly impacted by this limitation.

From 2000 to 2013, the hospital mortality rate for MV-ARDS showed a decreasing trend, dropping from 54.58% in 2000 to 43.41% in 2013. This improvement may be attributed to advancements in the treatment of etiological causes of ARDS and the implementation of lung-protective MV [9]. In their systematic review, Máca et al. [9] reported a hospital mortality rate of 45% from 2000 onwards, while Villar et al. [31] found a 53.2% hospital mortality rate for moderate-to-severe ARDS in Spain. Between 2014 and 2017, hospital mortality increased to rates exceeding 50%; this rise may be due to a detection bias, as fewer MV-ARDS cases were reported through the MBDS, probably because reported cases were the most severe patients. When the COVID-19 pandemic emerged and MV-ARDS incidence returned to normal values, mortality rate decreased to 45%. During 2021, the most severe year of the pandemic, the mortality rate further declined to below 40%, likely attributable to availability bias, as a higher number of MV-ARDS cases, including milder ones, were probably reported by clinicians. Studies indicate no significant difference in mortality between COVID-19-related ARDS and non-COVID-19-related ARDS patients [32], suggesting that no substantial variations in mortality rates could be expected, although COVID-19 had a significant impact on the absolute number of deaths due to ARDS [33]. The declining trend in hospital mortality observed in ARDS patients has also been noted among all critically ill patients [34], primarily attributed to improvements in quality of care [35, 36].

Boucher et al. [10] conducted a systematic review on costs associated with ARDS patients, and found that expenses could range from $8476 to $547,974 (2021 USD). In our study, MV-ARDS costs in Spain from 2000 to 2022 was in the range of €12,854 to €45,778, stabilizing at €30,000 to €40,000 in the last 6 years. This is comparable to the findings of McAuley et al. [37] in 2018 in the United Kingdom, where the cost per ARDS patient was £26,311 ± 20,162. This increase in health care costs cannot be fully explained by the general rise in prices in Spain, which was 68% between 2000 and 2022 [38], while ARDS healthcare costs increased by 100–200%. Generally, rising healthcare costs are linked to the rapid growth of the older population, greater longevity, and the increased illness burden among older patients [39]. Notably, the second period of our study shows a higher mean age compared to the first period. Moreover, LOHS was significantly longer during the second period of the study, with LOHS being a widely recognized factor contributing to higher healthcare costs [40]. In addition, nearly 19% of ARDS patients are readmitted within 30 days, which can incur an additional cost of approximately $27,000 per readmitted patient [41], which we could not assess in our study.

When comparing the pre-pandemic, intra-pandemic, and post-pandemic periods, we found that risk factors for severe COVID-19 infection were higher in MV-ARDS patients. These risk factors included male sex, older age, diabetes, and obesity [42]. In addition, aspergillosis was a prevalent co-infection during COVID-19 pandemic [43], while influenza infections decreased during that period [44]. In the intra-pandemic period, we observed a statistically significant longer duration of MV compared to the pre-pandemic and post-pandemic periods. This is consistent with existing evidence suggesting that COVID-19-associated ARDS is characterized by prolonged MV requirements [45, 46]. However, when comparing data between the ICD-9 and ICD-10 periods, interpretation may be confounded by the 20% of cases in the ICD-9 period with unspecified MV duration (96.70 code). This makes comparisons across coding systems challenging. Nevertheless, our findings add to the growing body of evidence that COVID-19 ARDS patients often require longer mechanical ventilation, reflecting the severity and unique pathophysiology of the disease.

Although administrative databases, such as the MBDS, contain a substantial amount of data, they have not been extensively used in studying ARDS epidemiology, with only two studies conducted in the US [47, 48] and one in Taiwan [49]. However, the accuracy of administrative coding remains a challenge for epidemiological studies on ARDS [6]. In our study, data collected for some years, particularly from 2013 to 2017, may be less representative due to a low number of MV-ARDS cases recorded in MBDS, likely attributable to coding transition and implementation of Berlin criteria for diagnosis. However, MBDS and national survey data offer a cost-saving approach for conducting research, and using these existing data sources to draw inferences that can inform future research objectives [50].

This study has limitations and strengths. First, as with any retrospective analysis, there is a possibility of under-coding of variables, leading to incomplete or inaccurate information. This could introduce potential bias and affect the robustness of our findings. Second, MBDS database lacked detailed data, including chest radiographs, arterial oxygen partial pressure to fractional inspired oxygen ratio (for grading ARDS severity). Third, since MBDS data are anonymous, it is impossible to determine whether a patient was hospitalized more than once in the same year, across multiple years, or in different hospitals. Fourth, the transition from ICD-9-CM to ICD-10-CM, along with the decline in cases make those years less representative. Following the recommendations of van Walraven et al. [51], we have addressed the issues posed by administrative databases to the best of our ability, acknowledging certain limitations. Fifth, although we only included patients on invasive mechanical ventilation, we mainly compared our results with studies that included the same kind of patients [26, 27, 31, 37]. The major strengths of this study include the large number of ARDS patients, which provides high statistical power and enhances the reliability of our analyses. In addition, the long follow-up period allows for a comprehensive assessment of trends over time. Moreover, the MBDS has an estimated coverage of 99.5% hospitals in Spain [13, 14], so the vast majority of hospitals and patients are recorded in this database, giving consistency to the data. To our knowledge, this is the largest nationwide epidemiological study of ARDS with the longest available follow-up period, offering a clear view of the disease’s trends.

## Conclusion

This is the largest nationwide epidemiological study of MV-ARDS with the longest follow-up period available in a European Country. Since 2000, the incidence of MV-ARDS in Spain has remained relatively stable, with some variation due to changes in coding and diagnostic criteria. Meanwhile, hospital mortality rates have steadily declined and stabilized around 40–45%. The cost of treating MV-ARDS has also risen nearly fourfold in recent years. During the COVID-19 pandemic, risk factors associated with COVID-19 were frequently observed in MV-ARDS cases. Utilizing MBDS data could greatly improve our understanding of ARDS and guide future research in this field.

## Supplementary Information

Additional file 1.

## Data Availability

The datasets used and/or analyzed during the current study are available from the corresponding author on reasonable request.

## Abbreviations

ARDS: Acute respiratory distress syndrome
DRG: Diagnosis-related groups
ICD: International Classification of Diseases
ICU: Intensive care unit
LOHS: Length of hospital stay
MBDS: Minimum basic dataset
MV: Mechanical ventilation
MV-ARDS: Mechanically ventilated acute respiratory distress syndrome
SD: Standard deviation
