# Supplementary material for: Epidemiological trends of mechanically ventilated acute respiratory distress syndrome in the twenty-first century: a nationwide, population-based retrospective study
Source: J Intensive Care. 2025 Feb 17;13:9. doi: 10.1186/s40560-025-00781-3 (PMC11831836; doi:10.1186/s40560-025-00781-3)
Supplement: Supplementary file 1 — Additional file 1. [file 40560_2025_781_MOESM1_ESM.docx]

**Supplementary table S1:** International Classification of Diseases, 9th Revision, Clinical Modification (ICD-9-CM) codes for sepsis diagnosis.

| **Organ System** | **ICD-9-CM Code** | **ICD-9-CM Code Description** |
| --- | --- | --- |
| **Nervous** |  |  |
|  | 013.* | Tuberculosis of meninges and central nervous system |
|  | 036.* | Meningococcal infection |
|  | 091.81 | Acute syphilitic meningitis (secondary) |
|  | 098.82 | Neurosyphilis |
|  | 320.* | Bacterial meningitis |
|  | 321.0 | Cryptococcal meningitis |
|  | 321.1 | Meningitis in other fungal diseases |
|  | 324.* | Central nervous system abscess |
|  | 325 | Phlebitis of intracranial sinus |
|  | 360.0 | Purulent endophthalmitis |
|  | 376.0 | Acute inflammation of orbit |
|  | 380.14 | Malignant otitis externa |
|  | 383.0.* | Acute mastoiditis |
| **Circulatory** |  |  |
|  | 093.* | Cardiovascular syphilis |
|  | 098.83 098.84 | Gonococcal infections |
|  | 036.4.* | Meningococcal carditis |
|  | 391.2 | Acute rheumatic myocarditis |
|  | 420.99 | Acute pericarditis due to other specified organisms |
|  | 421.* | Acute or subacute endocarditis |
| **Respiratory** |  |  |
|  | 010.1.* | Tuberculous pleurisy in primary progressive tuberculosis |
|  | 011.* | Pulmonary tuberculosis |
|  | 012.* | Other respiratory tuberculosis |
|  | 018.* | Miliary tuberculosis |
|  | 031.0 | Pulmonary diseases due to other mycobacteria |
|  | 032.* | Diphtheria |
|  | 034.* | Streptococcal throat/scarlet fever |
|  | 098.6 | Gonococcal infection of pharynx |
|  | 112.4 | Candidiasis, of lung |
|  | 114.0 | Primary coccidioidomycosis (pulmonary) |
|  | 114.1 | Primary extrapulmonary coccidioidomycosis |
|  | 115.15 | Histoplasma duboisii pneumonia |
|  | 115.05 | Histoplasma capsulatum pneumonia |
|  | 115.95 | Histoplasmosis pneumonia unspecified |
|  | 117.5 | Cryptococcus neoformans |
|  | 117.3 | Aspergillosis |
|  | 136.3 | Pneumocystosis |
|  | 461.* | Acute sinusitis |
|  | 462 | Acute pharyngitis |
|  | 463 | Acute tonsillitis |
|  | 464.* | Acute laryngitis/tracheitis |
|  | 465.* | Acute upper respiratory infection of multiple sites/not |
|  |  | otherwise specified |
|  | 475 | Peritonsillar abscess |
|  | 480.* | Viral pneumonia |
|  | 481 | Pneumococcal pneumonia |
|  | 482.* | Other bacterial pneumonia |
|  | 483.* | Pneumonia due to other specified organism |
|  | 485 | Bronchopneumonia with organism not otherwise specified |
|  | 486 | Pneumonia, organism not otherwise specified |
|  | 487.0 | Influenza with pneumonia |
|  | 487.1 | Influenza with other respiratory manifestations |
|  | 491.21 | Acute exacerbation of obstructive chronic bronchitis |
|  | 494.* | Bronchiectasis |
|  | 510.* | Empyema |
|  | 513.* | Abscess of lung and mediastinum |
| **Digestive** |  |  |
|  | 001.* | Cholera |
|  | 002.* | Typhoid/paratyphoid fever |
|  | 003.* | Other salmonella infection |
|  | 004.* | Shigellosis |
|  | 005.* | Other food poisoning |
|  | 008.0.* | Intestinal infections due to *Escherichia coli* |
|  | 008.1 | Intestinal infections due to Arizona group of paracolon bacillus |
|  | 008.2 | Intestinal infections due to *Aerobacter aerogenes* |
|  | 008.3 | Intestinal infections due to *Proteus* (*Mirabilis morganii*) |
|  | 008.4.* | Intestinal infections due to unspecified bacteria |
|  | 008.5 | Bacterial enteritis, unspecified |
|  | 009.* | Ill-defined intestinal infection |
|  | 014.* | Tuberculosis of intestines peritoneum and mesenteric glands |
|  | 129 | Intestinal parasitism unspecified |
|  | 522.5 | Periapical abscess without sinus |
|  | 522.7 | Periapical abscess with sinus |
|  | 526.4 | Inflammatory conditions of the jaw |
|  | 527.3 | Abscess of the salivary glands |
|  | 528.3 | Cellulitis and abscess of oral soft tissue |
|  | 540.* | Acute appendicitis |
|  | 541 | Appendicitis not otherwise specified |
|  | 542 | Other appendicitis |
|  | 562.01 | Diverticulitis of the small intestine without hemorrhage |
|  | 562.03 | Diverticulitis of the small intestine with hemorrhage |
|  | 562.11 | Diverticulitis of colon without hemorrhage |
|  | 562.13 | Diverticulitis of colon with hemorrhage |
|  | 566 | Abscess of the anal and rectal regions |
|  | 567.* | Peritonitis |
|  | 569.5 | Intestinal abscess |
|  | 569.61 | Infection of colostomy or enterostomy |
|  | 569.83 | Perforation of intestine |
|  | 572.0 | Abscess of liver |
|  | 572.1 | Portal pyemia |
|  | 575.0 | Acute cholecystitis |
| **Genitourinary** |  |  |
|  | 016.* | Tuberculosis of genitourinary system |
|  | 098.17 | Gonococcal salpingitis specified as acute |
|  | 112.2 | Candidiasis of other urogenital sites |
|  | 590.* | Kidney infection |
|  | 599.0 | Urinary tract infection not otherwise specified |
|  | 601.* | Prostatic inflammation |
|  | 604.* | Orchitis and epididymitis |
|  | 614.* | Female pelvic inflammation disease |
|  | 615.* | Uterine inflammatory disease |
|  | 616.3 | Abscess of Bartholin’s gland |
|  | 616.4 | Other abscess of vulva |
| **Pregnancy** |  |  |
|  | 634.0 | Spontaneous abortion, complicated by genital tract and pelvic infection |
|  |  | infection |
|  | 635.0 | Legally induced abortion, complicated by genital tract and |
|  |  | pelvic infection |
|  | 636.0 | Illegally induced abortion, complicated by genital tract and |
|  |  | pelvic infection |
|  | 637.0 | Unspecified abortion, complicated by genital tract and pelvic |
|  |  | infection |
|  | 638.0 | Failed attempted abortion, complicated by genital tract and |
|  |  | pelvic infection |
|  | 639.0 | Complications following abortion and ectopic and molar |
|  |  | pregnancies infection |
|  | 646.6.* | Infections of genitourinary tract in pregnancy |
|  | 658.4.* | Infection of amniotic cavity |
|  | 670.* | Major puerperal infection |
|  | 675.1.* | Abscess of breast |
| **Skin, soft tissue, or bone** | |  |
|  | 003.24 | Salmonella osteomyelitis |
|  | 015.* | Tuberculosis of bones and joints |
|  | 017.* | Tuberculosis of other organs |
|  | 031.1 | Cutaneous diseases due to other mycobacteria |
|  | 035 | Erysipelas |
|  | 036.82 | Meningococcal arthropathy |
|  | 040.0 | Gas gangrene |
|  | 095.5 | Syphilis of bone |
|  | 098.5.* | Gonococcal infection of joint |
|  | 681.* | Cellulitis, finger/toe |
|  | 682.* | Other cellulitis or abscess |
|  | 683 | Acute lymphadenitis |
|  | 685.0 | Pilonidal cyst, with abscess |
|  | 686.* | Other local skin infection |
|  | 711.0 | Pyogenic arthritis |
|  | 728.86 | Necrotizing fasciitis |
|  | 730.* | Osteomyelitis |
| **Other** |  |  |
|  | 790.7 | Bacteremia |
|  | 958.3 | Posttraumatic wound infection, not elsewhere classified |
|  | 996.6.* | Infection or inflammation of device/graft |
|  | 998.5.* | Postoperative infection |
|  | 999.3.* | Infectious complication of medical care not otherwise classified |

**Supplementary table S2:** International Classification of Diseases, 10th Revision, Clinical Modification (ICD-10-CM) codes for sepsis diagnosis.

| A02.1 | Salmonella septicemia |
| --- | --- |
| A20.7 | Septicemic plague |
| A22.7 | Septicemia due to anthrax |
| A39.4 | Meningococcal septicemia |
| A39.1 | Waterhouse-Friderichsen syndrome |
| A41.2 | Staphylococcal, unspecified |
| A41.0 | Sepsis due to Staphylococcus aureus |
| A41.1 | Sepsis due to other specified staphylococci |
| A40.* | Streptococcal sepsis |
| A41.4 | Sepsis due to anaerobes |
| A41.50 | Sepsis due to other gram-negative organisms NEOM |
| A41.59 | Sepsis due others Gram negatives |
| A41.3 | Sepsis due to Haemophilus influenzae |
| A41.51 | Escherichia coli sepsis [E. coli] |
| A41.52 | Pseudomonas sepsis |
| A41.53 | Sepsis due to Serratia |
| A41.59 | Sepsis due to other gram negatives |
| A41.89 | Other specified sepsis |
| A41.9 | Sepsis, unspecified organism (septicemia NEOM) |
| A54.86 | Gonococcal septicemia |
| A48.3 | Toxic shock syndrome |
| B37.7 | Candida sepsis |
| B37.6 | Candidal endocarditis |
| B38.7 | Disseminated coccidioidomycosis |
| N39.0  B37.4.*  N30.*  N34.*  R82.81 | Urinary tract infection |
| R65.* | Symptoms and signs specifically associated with systemic inflammation and infection |
| R78.81 | Bacteremia |
| T80.89 | Complication of medical care, other transfusion reaction |
| T81.1.* | Postprocedural shock |
| T81.4.* | Infection following a procedure |
| R65.21 | Shock septic |
| B37.5 | Candidal meningitis |
| B37.6 | Candidal endocarditis |
| B37.7 | Candidal sepsis |
| B37.8 | Candidiasis of other sites |
| B37.9 | Candidiasis, unspecified |
| B44.0 | Invasive pulmonary aspergillosis |
| B44.1 | Other pulmonary aspergillosis |
| B44.2 | Tonsillar aspergillosis |
| B44.7 | Disseminated aspergillosis |
| B44.8 | Other forms of aspergillosis |
| B44.9 | Aspergillosis, unspecified |
| B45.0 | Pulmonary cryptococcosis |
| B45.1 | Cerebral cryptococcosis |
| B45.2 | Cutaneous cryptococcosis |
| B45.3 | Osseous cryptococcosis |
| B45.7 | Disseminated cryptococcosis |
| B45.8 | Other forms of cryptococcosis |
| B45.9 | Cryptococcosis, unspecified |
| B46.0 | Pulmonary mucormycosis |
| B46.1 | Rhinocerebral mucormycosis |
| B46.2 | Gastrointestinal mucormycosis |
| B46.3 | Cutaneous mucormycosis |
| B46.4 | Disseminated mucormycosis |
| B46.5 | Mucormycosis, unspecified |
| B46.8 | Other zygomycoses |
| B46.9 | Zygomycosis, unspecified |
| B49 | Unspecified mycosis |

**Supplementary Table S3**: International Classification of Diseases, 9th Revision, Clinical Modification (ICD-9-CM) codes for acute organ dysfunction.

| **Organ System** | **ICD-9-CM Code** | **ICD-9-CM Code Description** |
| --- | --- | --- |
| Cardiovascular | 427.5 | Cardiac arrest |
|  | 458.0 | Orthostatic hypotension |
|  | 458.8 | Other specified hypotension |
|  | 458.9 | Hypotension, unspecified |
|  | 785.5.* | Shock without mention of trauma |
|  | 796.3 | Hypotension, transient |
| Hematologic | 286.2 | Disseminated intravascular coagulation |
|  | 286.6 | Defibrination syndrome |
|  | 286.9 | Other and unspecified coagulation defects |
|  | 287.3.*  287.4.*  287.5 | Thrombocytopenia, primary, secondary or unspecified |
|  | 790.92 | Abnormal coagulation profile |
| Hepatic | 570 | Acute and subacute necrosis of liver |
|  | 572.2 | Hepatic encephalopathy |
|  | 573.3 | Hepatitis (septic & not elsewhere classified) |
|  | 573.4 | Hepatic infarction |
| Neurologic | 293.* | Transient organic psychosis |
|  | 348.1 | Anoxic brain damage |
|  | 348.3.* | Encephalopathy, acute |
|  | 780.01 | Coma |
|  | 780.09 | Altered consciousness, unspecified |
|  | 89.14 | Electroencephalography |
| Renal | 580.* | Acute glomerulonephritis |
|  | 584.* | Acute renal failure |
|  | 586 | Renal shutdown, renal failure unspecified |
|  | 39.95 | Hemodialysis |
| Respiratory | 518.5.* | Pulmonary insufficiency following trauma and surgery |
|  | 518.8.* | Respiratory failure |
|  | 786.03 | Apnea |
|  | 799.1 | Respiratory arrest |
|  | 786.09 | Respiratory insufficiency |
|  | 96.7.* | Ventilator management |
|  | 96.04 | Endotracheal intubation (emergency procedure) |
|  | 93.90 | Continuous positive airway pressureº |
| Metabolic | 276.2 | Acidosis, metabolic or lactic |

**Supplementary Table S4**: International Classification of Diseases, 10th Revision, Clinical Modification (ICD-10-CM) codes for acute organ dysfunction.

| **Organ System** | **ICD-10-CM Code** | **ICD-10-CM Code Description** |
| --- | --- | --- |
| **Cardiovascular** | I46 | Cardiac arrest |
|  | I95.1 | Orthostatic hypotension |
|  | I95.89 | Other specified hypotension |
|  | I95.9 | Hypotension, unspecified |
|  | R57.0 | Shock without mention of trauma |
|  | R57.9 | Hypotension, transient |
| **Hematologic** | D65 | Disseminated intravascular coagulation (Defibrination syndrome) |
|  | D68 | Other and unspecified coagulation defects |
|  | D69 | Purpura and other bleeding conditions |
|  | R79.1 | Abnormal coagulation profile |
| **Hepatic** | K72.0 | Acute and subacute necrosis of liver |
|  | K72.01, K72.91 | Hepatic encephalopathy |
|  | K75.9 | Hepatitis (septic & not elsewhere classified) |
|  | K76.3 | Hepatic infarction |
| **Neurologic** | F06 | Transient organic psychosis |
|  | G93.1 | Anoxic brain damage |
|  | G93.4 | Other and unspecified types of encephalopathy |
|  | G31.2 | Alcoholic encephalopathy |
|  | G94 | Other disorders of the brain in diseases classified elsewhere |
|  | I67.4 | Hypertensive encephalopathy |
|  | R40 | Drowsiness, stupor and coma |
|  | 4A00 | Electroencephalography |
| **Renal** | N00 | Acute glomerulonephritis |
|  | N17 | Acute renal failure |
|  | N19 | Renal shutdown, renal failure unspecified |
|  | 5A1D | Hemodialysis |
| **Respiratory** | J95 | Intraoperative and postprocedural complications and disorders of the respiratory system, not elsewhere classified |
|  | R06.81 | Apnea |
|  | R09.2 | Respiratory arrest |
|  | J96 | Respiratory insufficiency |
|  | 5A19, 5A09 | Ventilator management |
|  | [0BH1](javascript:loadCT(%220BH1%22,%22cie10pcs%22,%222012%22)) | Endotracheal intubation (emergency procedure) |
|  | A09357, 5A09557, 5A09457 | Continuous positive airway pressure |
| **Metabolic** | E87.2 | Acidosis, metabolic or lactic |

**Supplementary Table S5**: International Classification of Diseases, 9th Revision, Clinical Modification (ICD-9-CM) codes for the site of infection.

| **Organ System** | **ICD-9-CM Code** | **ICD-9-CM Code Description** |
| --- | --- | --- |
| **Nervous** |  |  |
|  | 013.* | Tuberculosis of meninges and central nervous system |
|  | 036.* | Meningococcal infection |
|  | 091.81 | Acute syphilitic meningitis (secondary) |
|  | 098.82 | Neurosyphilis |
|  | 320.* | Bacterial meningitis |
|  | 321.0 | Cryptococcal meningitis |
|  | 321.1 | Meningitis in other fungal diseases |
|  | 324.* | Central nervous system abscess |
|  | 325 | Phlebitis of intracranial sinus |
|  | 360.0 | Purulent endophthalmitis |
|  | 376.0 | Acute inflammation of orbit |
|  | 380.14 | Malignant otitis externa |
|  | 383.0.* | Acute mastoiditis |
| **Circulatory** |  |  |
|  | 093.* | Cardiovascular syphilis |
|  | 098.83 098.84 | Gonococcal infections |
|  | 036.4.* | Meningococcal carditis |
|  | 391.2 | Acute rheumatic myocarditis |
|  | 420.99 | Acute pericarditis due to other specified organisms |
|  | 421.* | Acute or subacute endocarditis |
| **Respiratory** |  |  |
|  | 010.1.* | Tuberculous pleurisy in primary progressive tuberculosis |
|  | 011.* | Pulmonary tuberculosis |
|  | 012.* | Other respiratory tuberculosis |
|  | 018.* | Miliary tuberculosis |
|  | 031.0 | Pulmonary diseases due to other mycobacteria |
|  | 032.* | Diphtheria |
|  | 034.* | Streptococcal throat/scarlet fever |
|  | 098.6 | Gonococcal infection of pharynx |
|  | 112.4 | Candidiasis, of lung |
|  | 114.0 | Primary coccidioidomycosis (pulmonary) |
|  | 114.1 | Primary extrapulmonary coccidioidomycosis |
|  | 115.15 | Histoplasma duboisii pneumonia |
|  | 115.05 | Histoplasma capsulatum pneumonia |
|  | 115.95 | Histoplasmosis pneumonia unspecified |
|  | 117.5 | Cryptococcus neoformans |
|  | 117.3 | Aspergillosis |
|  | 136.3 | Pneumocystosis |
|  | 461.* | Acute sinusitis |
|  | 462 | Acute pharyngitis |
|  | 463 | Acute tonsillitis |
|  | 464.* | Acute laryngitis/tracheitis |
|  | 465.* | Acute upper respiratory infection of multiple sites/not |
|  |  | otherwise specified |
|  | 475 | Peritonsillar abscess |
|  | 480.* | Viral pneumonia |
|  | 481 | Pneumococcal pneumonia |
|  | 482.* | Other bacterial pneumonia |
|  | 483.* | Pneumonia due to other specified organism |
|  | 485 | Bronchopneumonia with organism not otherwise specified |
|  | 486 | Pneumonia, organism not otherwise specified |
|  | 487.0 | Influenza with pneumonia |
|  | 487.1 | Influenza with other respiratory manifestations |
|  | 491.21 | Acute exacerbation of obstructive chronic bronchitis |
|  | 494.* | Bronchiectasis |
|  | 510.* | Empyema |
|  | 513.* | Abscess of lung and mediastinum |
| **Digestive** |  |  |
|  | 001.* | Cholera |
|  | 002.* | Typhoid/paratyphoid fever |
|  | 003.* | Other salmonella infection |
|  | 004.* | Shigellosis |
|  | 005.* | Other food poisoning |
|  | 008.0.* | Intestinal infections due to *Escherichia coli* |
|  | 008.1 | Intestinal infections due to Arizona group of paracolon bacillus |
|  | 008.2 | Intestinal infections due to *Aerobacter aerogenes* |
|  | 008.3 | Intestinal infections due to *Proteus* (*Mirabilis morganii*) |
|  | 008.4.* | Intestinal infections due to unspecified bacteria |
|  | 008.5 | Bacterial enteritis, unspecified |
|  | 009.* | Ill-defined intestinal infection |
|  | 014.* | Tuberculosis of intestines peritoneum and mesenteric glands |
|  | 129 | Intestinal parasitism unspecified |
|  | 522.5 | Periapical abscess without sinus |
|  | 522.7 | Periapical abscess with sinus |
|  | 526.4 | Inflammatory conditions of the jaw |
|  | 527.3 | Abscess of the salivary glands |
|  | 528.3 | Cellulitis and abscess of oral soft tissue |
|  | 540.* | Acute appendicitis |
|  | 541 | Appendicitis not otherwise specified |
|  | 542 | Other appendicitis |
|  | 562.01 | Diverticulitis of the small intestine without hemorrhage |
|  | 562.03 | Diverticulitis of the small intestine with hemorrhage |
|  | 562.11 | Diverticulitis of colon without hemorrhage |
|  | 562.13 | Diverticulitis of colon with hemorrhage |
|  | 566 | Abscess of the anal and rectal regions |
|  | 567.* | Peritonitis |
|  | 569.5 | Intestinal abscess |
|  | 569.61 | Infection of colostomy or enterostomy |
|  | 569.83 | Perforation of intestine |
|  | 572.0 | Abscess of liver |
|  | 572.1 | Portal pyemia |
|  | 575.0 | Acute cholecystitis |
| **Genitourinary** |  |  |
|  | 016.* | Tuberculosis of genitourinary system |
|  | 098.17 | Gonococcal salpingitis specified as acute |
|  | 112.2 | Candidiasis of other urogenital sites |
|  | 590.* | Kidney infection |
|  | 599.0 | Urinary tract infection not otherwise specified |
|  | 601.* | Prostatic inflammation |
|  | 604.* | Orchitis and epididymitis |
|  | 614.* | Female pelvic inflammation disease |
|  | 615.* | Uterine inflammatory disease |
|  | 616.3 | Abscess of Bartholin’s gland |
|  | 616.4 | Other abscess of vulva |
| **Pregnancy** |  |  |
|  | 634.0 | Spontaneous abortion, complicated by genital tract and pelvic infection |
|  |  | infection |
|  | 635.0 | Legally induced abortion, complicated by genital tract and |
|  |  | pelvic infection |
|  | 636.0 | Illegally induced abortion, complicated by genital tract and |
|  |  | pelvic infection |
|  | 637.0 | Unspecified abortion, complicated by genital tract and pelvic |
|  |  | infection |
|  | 638.0 | Failed attempted abortion, complicated by genital tract and |
|  |  | pelvic infection |
|  | 639.0 | Complications following abortion and ectopic and molar |
|  |  | pregnancies infection |
|  | 646.6.* | Infections of genitourinary tract in pregnancy |
|  | 658.4.* | Infection of amniotic cavity |
|  | 670.* | Major puerperal infection |
|  | 675.1.* | Abscess of breast |
| **Skin, soft tissue, or bone** | |  |
|  | 003.24 | Salmonella osteomyelitis |
|  | 015.* | Tuberculosis of bones and joints |
|  | 017.* | Tuberculosis of other organs |
|  | 031.1 | Cutaneous diseases due to other mycobacteria |
|  | 035 | Erysipelas |
|  | 036.82 | Meningococcal arthropathy |
|  | 040.0 | Gas gangrene |
|  | 095.5 | Syphilis of bone |
|  | 098.5.* | Gonococcal infection of joint |
|  | 681.* | Cellulitis, finger/toe |
|  | 682.* | Other cellulitis or abscess |
|  | 683 | Acute lymphadenitis |
|  | 685.0 | Pilonidal cyst, with abscess |
|  | 686.* | Other local skin infection |
|  | 711.0 | Pyogenic arthritis |
|  | 728.86 | Necrotizing fasciitis |
|  | 730.* | Osteomyelitis |
| **Other** |  |  |
|  | 790.7 | Bacteremia |
|  | 958.3 | Posttraumatic wound infection, not elsewhere classified |
|  | 996.6.* | Infection or inflammation of device/graft |
|  | 998.5.* | Postoperative infection |
|  | 999.3.* | Infectious complication of medical care not otherwise classified |

**Supplementary Table S6**: International Classification of Diseases, 10th Revision, Clinical Modification (ICD-10-CM) codes for the site of infection.

|  | **Nervous** |
| --- | --- |
| A17 | Tuberculosis of meninges and central nervous system |
| A39.0 | Meningococcal infection |
| A51.41 | Acute syphilitic meningitis (secondary) |
| A52.1 | symptomatic neurosyphilis |
| A52.2 | Asymptomatic neurosyphilis |
| A52.3 | Unspecified neurosyphilis |
| G00 | Bacterial meningitis, not elsewhere classified |
| G04.2 | Bacterial meningoencephalitis and meningomyelitis, not elsewhere classified |
| B45.1 | Cerebral cryptococcosis |
| G02 | Meningitis in other infectious and parasitic diseases classified elsewhere |
| G06 | Central nervous system abscess |
| G08 | Phlebitis of intracranial sinus |
| H44.0 | Purulent endophthalmitis |
| H05.00 | Acute inflammation of orbit |
| H60.20 | Malignant otitis externa |
| H70.0 | Acute mastoiditis |
|  | **Circulatory** |
| A52.00-A52.04 | Cardiovascular syphilis |
| A52.06 | Cardiovascular syphilis |
| A52.09 | Cardiovascular syphilis |
| A54.83 | gonococcal heart infection (endocarditis, pericarditis, myocarditis) |
| I01.2 | Acute rheumatic myocarditis |
| I30 | Acute pericarditis |
| I33 | Acute or subacute endocarditis |
|  | **Respiratory** |
| A15 | respiratory tuberculosis |
| A31.0 | Pulmonary diseases due to other mycobacteria |
| A36 | Diphtheria |
| A38 | Streptococcal throat/scarlet fever |
| A54.5 | Gonococcal infection of pharynx |
| B37.1 | Candidiasis, of lung |
| B38.0 | Acute pulmonary coccidioidomycosis |
| B38.1 | Chronic pulmonary coccidioidomycosis |
| B39.5 | Histoplasma duboisii pneumonia |
| B39.2 | Histoplasma capsulatum pneumonia |
| B39.9 | Histoplasmosis pneumonia unspecified |
| B45 | Cryptococcus neoformans |
| B44 | Aspergillosis |
| B59 | Pneumocystosis |
| J01 | Acute sinusitis |
| J02 | Acute pharyngitis |
| J03 | Acute tonsillitis |
| J04 | Acute laryngitis/tracheitis |
| J06 | Acute upper respiratory infection of multiple sites/not |
| J36 | Peritonsillar abscess |
| J12 | Viral pneumonia |
| J13 | Pneumococcal pneumonia |
| J15 | Other bacterial pneumonia |
| J16 | Pneumonia due to another specified organism |
| J18 | Pneumonia, organism not otherwise specified |
| J10 | Influenza |
| J44.1 | Acute exacerbation of obstructive chronic bronchitis |
| J47 | Bronchiectasis |
| J86 | Pyothorax |
| J85 | Abscess of lung and mediastinum |
|  | **Digestive** |
| A00 | Cholera |
| A01 | Typhoid/paratyphoid fever |
| A02 | Other salmonella infection |
| A03 | Shigellosis |
| A05 | Other food poisoning |
| A04.0-A04.4 | Intestinal infections due to *Escherichia coli* |
| A04.8 | Other specified bacterial intestinal infections |
| A04.9 | Bacterial intestinal infection, unspecified |
| A18.3 | Tuberculosis of intestines peritoneum and mesenteric glands |
| B82 | Intestinal parasitism unspecified |
| K04.7 | Periapical abscess without sinus |
| K04.6 | Periapical abscess with sinus |
| M27.2 | Inflammatory conditions of the jaw |
| K11.3 | Abscess of the salivary glands |
| K12.2 | Cellulitis and abscess of oral soft tissue |
| K35 | Acute appendicitis |
| K37 | Appendicitis not otherwise specified |
| K36 | Other appendicitis |
| K57.12 | Diverticulitis of the small intestine without haemorrhage |
| K57.13 | Diverticulitis of the small intestine with haemorrhage |
| K57.32 | Diverticulitis of colon without haemorrhage |
| K57.33 | Diverticulitis of colon with haemorrhage |
| K61.0 | Anal abscess |
| K61.1 | rectal abscess |
| K61.3 | Ischiorectal abscess |
| K65 | Peritonitis |
| K63.0 | Intestinal abscess |
| K94.02 | Infection of colostomy |
| K94.12 | Infection of enterostomy |
| K63.1 | Perforation of intestine |
| K75.0 | Abscess of liver |
| K75.1 | Portal pyaemia |
| K81.0 | Acute cholecystitis |
|  | **Genitourinary** |
| A18.1 | Tuberculosis of genitourinary system |
| A54.24 | Gonococcal salpingitis specified as acute |
| B37.4 | Candidiasis of other urogenital sites |
| N10 | Kidney infection |
| N39.0 | Urinary tract infection not otherwise specified |
| N41 | Prostatic inflammation |
| N45 | Orchitis and epididymitis |
| N73 | Female pelvic inflammation disease |
| N71 | Uterine inflammatory disease |
| N75.1 | Abscess of Bartholin’s gland |
| N76.4 | Other abscess of vulva |
|  | **Pregnancy** |
| O03 | Spontaneous abortion |
| O04 | Complications after (induced) termination of pregnancy |
| O08 | Complications following abortion and ectopic and molar pregnancies |
| O23 | Infections of genitourinary tract in pregnancy |
| O41.1 | Infection of amniotic cavity |
| O85 | Major puerperal infection |
| N61.1 | Abscess of breast |
|  | **Skin, soft tissue, or bone** |
| A02.24 | Salmonella osteomyelitis |
| A18.0 | Tuberculosis of bones and joints |
| A18 | Tuberculosis of other organs |
| A31.1 | Cutaneous diseases due to other mycobacteria |
| A146 | Erysipelas |
| A39.83 | Meningococcal arthropathy |
| A48.0 | Gas gangrene |
| A52.77 | Syphilis of bone |
| A54.5 | Gonococcal infection of joint |
| L03 | Cellulitis and acute lymphangitis |
| L05.01 | Pilonidal cyst, with abscess |
| L08 | Other local skin infection |
| M00 | Pyogenic arthritis |
| M72.6 | Necrotizing fasciitis |
| M86 | Osteomyelitis |
|  | **Other** |
| R78.81 | Bacteraemia |
| T79.8XXA | Post-traumatic wound infection, not elsewhere classified |
| T82.7 | Infection or inflammation of device/graft |
| T81. 4 | Postoperative infection |

**Supplementary table S7.** International Classification of Diseases, 10th Revision, Clinical Modification (ICD-9-CM) codes for Charlson Index calculation.

| **Comorbidities** | **ICD-9-CM** |
| --- | --- |
| Myocardial infarction | 410.*, 412.* |
| Congestive heart failure | 428.* |
| Peripheral vascular disease | 443.9, 441.*, 785.4, V43.4, Procedure 38.48 |
| Cerebrovascular disease | 430.*, 431.*,432, 433.*, 434.*, 435.*, 436.*, 437.*, 438.* |
| Dementia | 290.* |
| Chronic pulmonary disease | 490.*–505.x*, 506.4 |
| Rheumatic disease | 710.0, 710.1, 710.4, 714.0–714.2, 714.81, 725.x |
| Peptic ulcer disease | 531.*–534.* |
| Mild liver disease | 571.2, 571.4.*, 571.5, 571.6 |
| Diabetes without chronic complication | 250.0.*, 250.1.*, 250.2.*, 250.3.*, 250.7.* |
| Diabetes with chronic complication | 250.4.*, 240.5.*, 250.6.* |
| Hemiplegia or paraplegia | 344.1, 342.* |
| Renal disease | 582.*, 583.0, 583.1, 583.2, 583.4, 583.6, 583.7, 585.*, 586.*, 588.* |
| Any malignancy, including lymphoma and leukemia, except malignant neoplasm of skin | 140.*–172.*, 174.*.–195.8, 200.*–208.* |
| Moderate or severe liver disease | 456.0–456.21, 572.2–572.8 |
| Metastatic solid tumor | 196.*–199.1 |
| AIDS/HIV | 042.* |

**Supplementary table S8.** International Classification of Diseases, 10th Revision, Clinical Modification (ICD-10-CM) codes for Charlson Index calculation.

| Myocardial infarction | I21.*  I22.*  I25.2 |
| --- | --- |
| Congestive heart failure | I09.9  I11.0  I13.0  I13.2  I25.5  I42.0  I42.5  I42.6  I42.7  I42.8  I42.9  I43  I50.* |
| Peripheral vascular disease | I70.*  I71.*  I67.1  I73.1  I73.8.*  I73.9  I77.1  I79.0  K55.1  K55.8  K55.9  Z95.8.*  Z95.9 |
| Cerebrovascular disease | G45,*  G46.*  H34.*  I60.*  I61.*  I62.*  I63.*  I65.*  I66.*  I67.*  I68.*  I69.* |
| Dementia | F01.*  F02.*  F03.*  G30.*  G31.1 |
| Chronic pulmonary disease | I27.8  I27.9  J40.*  J41.*  J42.*  J43.*  J44.*  J45.*  J47.*  J60.*  J61.*  J62.*  J63.*  J64.*  J65.*  J66.*  J67.*  J68.4  J70.1  J70.3 |
| Rheumatic disease | M05.*  M06.*  M31.5  M32.*  M33.*  M34.*  M35.1  M35.3  M36.0 |
| Peptic ulcer disease | K25.*  K26.*  K27.*  K28.* |
| Mild liver disease | B18.*  K70.0  K70.1.*  K70.2  K70.3.*  K70.9  K71.3  K71.4  K71.5.*  K71.7  K73.*  K74.*  K76.0  K76.2  K76.3  K76.4  K76.8.*  K76.9  Z94.4 |
| Diabetes without chronic complication | E10.1.*  E10.6.*  E10.9  E11.0.*  E11.1.*  E11.6.*  E11.8  E11.9  E13.0.*  E13.1.*  E13.6.*  E13.8  E13.9 |
| Diabetes with chronic complication | E10.2.*  E10.3.*  E10.4.*  E10.5.*  E11.2.*  E11.3.*  E11.4.*  E11.5.*  E13.2.*  E13.3.*  E13.4.*  E13.5.* |
| Paraplegia and hemiplegia | G80.*  G81.*  G82.*  G04.1  G11.4  G83.0  G83.1.*  G83.2.*  G83.3.*  G83.4  G83.9.* |
| Renal disease | N18.*  N19.*  N05.2  N05.3  N05.4  N05.5  N05.6  N05.7  N25.0  I12.0  I13.1.*  N03.2  N03.3  N03.4  N03.5  N03.6  N03.7  Z49.*  Z94.0  Z99.2 |
| Any malignancy, including lymphoma and leukaemia, except malignant neoplasm of skin | C00.*-C26.*  C30.*-C34.*  C37.*-C41.*  C43.*  C45.*-C58.*  C60.*-C76.*  C81.*-C85.*  C88.*  C90.*-C97.* |
| Moderate or severe liver disease | K70.4  K71.1.*  K72.1.*  K72.9.*  K76.5  K76.6  K76.7  I85.0.*  I86.4  I98.2 |
| Metastatic carcinoma | C77.*  C78.*  C79.*  C80.* |
| AIDS/HIV | B20.* |
